# Development of Alginate/Carboxymethylcellulose Films Incorporated with *Canavalia ensiformis* Lectin (ConA) with Angiogenic Properties

**DOI:** 10.1021/acsomega.5c05146

**Published:** 2025-11-03

**Authors:** Maria Helena C. Santos, Ana Lúcia E. Santos, Israel J. M. Santos, Renato R. Roma, Abel V. M. Bisneto, Clever G. Cardoso, Bruno A. M. Rocha, Lee Chen-Chen, Aryane Tofanello, Wanius Garcia, Luís C. N. Silva, Ariane M. S. Santos, Edson C. Silva-Filho, Claudener S. Teixeira

**Affiliations:** † Department of Biochemistry and Molecular Biology, Federal University of Ceará, Fortaleza, Ceará 60451-970, Brazil; ‡ Center for Agricultural Sciences and Biodiversity, Federal University of Cariri, Crato, Ceará 63130-025, Brazil; § Department of Biological Chemistry, Regional University of Cariri, Crato, Ceará 63105-000, Brazil; ∥ Department of General Biology, Federal University of Goiás, Goiânia, Goiás 74001-970, Brazil; ⊥ Center for Natural and Human Sciences, Federal University of ABC, Santo André, São Paulo 09210-580, Brazil; # Laboratory of Microbial Pathogenesis, 125287 CEUMA University, São Luís 65045-380, Brazil; ¶ Interdisciplinary Laboratory of Advanced Materials, Federal University of Piauí, Teresina, Piauí 64049-550, Brazil

## Abstract

The development of new materials for wound care is a critical area, focused on creating dressings with improved properties, such as high absorption, flexibility, and low cost. In this context, natural polymers such as alginate and carboxymethyl cellulose (CMC) emerge as promising choices, given their biodegradability and their ability to promote an ideal healing environment. Concomitantly, lectins with angiogenic potential have been extensively investigated for their ability to modulate cellular responses and induce the formation of new blood vessels. This research aims to incorporate the lectin from *Canavalia ensiformis* (ConA) into alginate and carboxymethylcellulose (CMC) films to promote blood vessel growth and induce revascularization as a therapeutic approach. Film characterization and physicochemical tests showed efficient lectin/film incorporation, as observed through differential scanning calorimetry (DSC) analysis and Fourier-transform infrared spectroscopy (FTIR). DSC analysis showed that alginate/CMC films with ConA tend to retain less water, volatilizing more easily, with a temperature difference of 94 and 81 °C to 69 and 77 °C films containing ConA. In addition to the significantly prolonged retention capacity of ConA in the film, FTIR data suggest that ConA is anchored in the alginate matrix due to the cross-linked nature of the film chain, with minimal chemical interactions (or chemical bonds). Analysis of hemagglutinating activity and immunohistochemical assays showed an increase in the expression of the angiogenic factors TGF-β and VEGF. These results indicate that the biopolymers used are an effective alternative for wound treatment, pointing to future research into the development of therapeutic biofilms.

## Graphical abstract

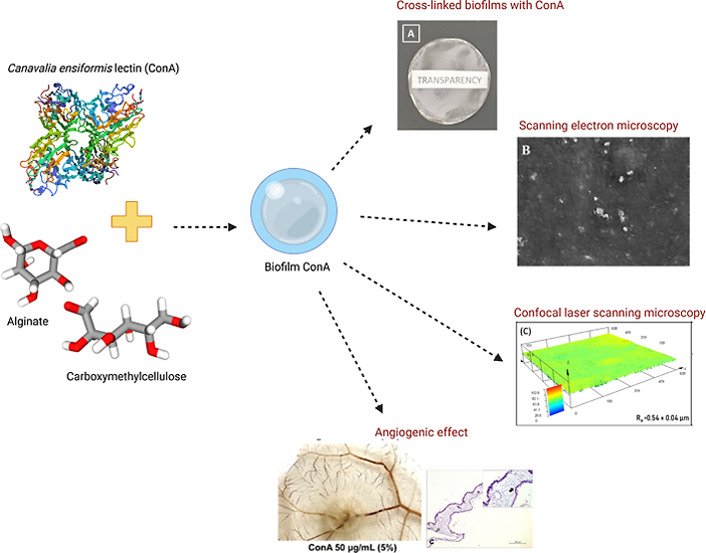

## 1 Introduction

Angiogenesis is defined as the formation of new blood vessels from the endothelial cells (ECs) of pre-existing veins, arteries, and capillaries. It plays an essential role in embryogenesis and during life through physiological tissue development and repair processes.^1^

Endogenous chemical signals, local or systemic, coordinate the functions of endothelial cells and smooth muscle cells to repair damaged blood vessels,^2^ which is a potentially beneficial approach in conditions where the promotion of new vessels can improve the perfusion of compromised tissues.^3^ At the cellular level, degradation of the extracellular matrix increases the concentration of various growth factors, stimulating the migration and proliferation of endothelial cells. The formation of the vascular network consists of multiple coordinated, sequential, and interdependent steps mediated by various angiogenic factors, including growth factors, chemokines, angiogenic enzymes, endothelial-specific receptors, and adhesion molecules.^4^ In the process, the transforming growth factor β family (TGF-β, including TGF-β 1, TGF-β 2, and TGF-β 3), cytokines, and VEFG family play crucial roles.^5^

Hydrogel films are an important class of materials used in various biomedical, pharmaceutical, and environmental applications.^6,7^ Some hydrogels are combined with alginate and sodium carboxymethyl cellulose (CMC) due to their biocompatibility, biodegradability, low cost, and ease of production.^8^ Because they contain a high percentage of water, hydrogels act by softening and removing devitalized tissue through debridement tissue through autolytic debridement. The water keeps the environment moist, and carboxymethylcellulose facilitates cellular rehydration and debridement. In addition, hydrogel films can serve as vehicles for bioactive molecules, including lectins.^9,10^

Concanavalin A (ConA) is one of the most well-characterized lectins, both in terms of its three-dimensional structure and its biological applications, which are based on its recognition and interaction with glycans present on the surface of various cells.^11^ Among its key biological applications are the modulation of antibiotic activity in resistant bacteria,^12^ neuroprotection,^13^ leishmanicidal^14^ and fungicidal activity,^15^ among others.

Furthermore, studies such as that by Vale de Macedo et al.,^16^ which used alginate and glycerol films conjugated with ConA and gentamicin, have shown promise for the treatment of skin lesions. These works demonstrated effective antimicrobial activity and encouraged subsequent in vivo investigations into healing properties.

ConA-like lectins, such as *Dioclea violacea* lectin (DVL) and *Vatairea macrocarpa* lectin (VML), have attracted increasing interest due to their remarkable angiogenic effect.^17^ These proteins, abundantly present in plants, have been shown to positively influence the formation of new blood vessels in various contexts.^18,19^ The interaction between lectins and endothelial cells has been associated with the modulation of growth factors and cytokines, thus promoting angiogenesis and contributing to tissue regenerative processes.^20^ However, none of these studies has evaluated the controlled release of these lectins during angiogenesis. This represents a new strategy that could lead to the development of a new class of materials with applications in wound healing.

As a whole, the research highlights the feasibility of integrating the ConA lectin with a film composed of alginate and carboxymethylcellulose (CMC), inducing angiogenesis pathways and promoting vascularization, which is essential in the advancement of therapeutic approaches and innovative drugs.

## 2 Methods

### 2.1 Chemicals

The reagents used in the tests are of analytical quality. For lectin purification, reagents from Sigma-Aldrich were used. For the production of films, sodium alginate (Cat No. W201502), carboxymethylcellulose (CMC) (Cat No. 21904) and calcium chloride (Cat No. P.10.0294.021.00.27) were from Dinâmica. Tris-HCl (Cat No. 10812846001) was obtained from Sigma-Aldrich. NaCl was purchased from Química Moderna (Brazil) (Cat No. QMA00001145700500). For angiogenic assessment assays, all reagents were obtained from Sigma-Aldrich, unless otherwise specified. Dersani healing oil was purchased from Laboratório Doudt Oliveira Ltda. The staining solutions (hematoxylin and eosin) were purchased from LaborClin Ltda. All chemicals and reagents used in this study were of the highest purity and analytical grade.

### 2.2 Purification, Production, and Characterization of Films

#### 2.2.1 Purification of *Canavalia ensiformis* Seeds—ConA

*C. ensiformis* seeds were collected from plants located in Crato, Ceará, Brazil, the seed collection was registered in SISGEN (Genetic Heritage and Associated Traditional Knowledge Management System, ID: AF8E1DD). The seeds of *C. ensiformis* were ground to obtain a fine powder using an electric coffee mill, the soluble proteins were extracted at 25 °C by continuous stirring with 50 mL of 0.15 mol/L NaCl in 5 g of powder for 4 h, followed by centrifugation at 10,000*g* 4 °C for 20 min. Protein purification was carried out using the affinity chromatography in a Sephadex-G50 column (Sigma, Saint Louis, USA) (2 × 20 cm) and eluted with 0.1 mol/L glucose. The fraction containing lectin from *C. ensiformis* (ConA) was then lyophilized and its homogeneity assessed by sodium dodecyl sulfate-polyacrylamide gel electrophoresis (SDS-PAGE) (Supporting Information Figure S1), following the method described by Laemmli.^21^

#### 2.2.2 Formulation of a Cross-Linked Alginate + Film Carboxymethylcellulose Incorporated with the ConA Lectin

The preparation and consecutive production of films based on alginate and carboxymethylcellulose (CMC) was achieved with two stages of cross-linking with calcium ions using the casting method.

Initially, solutions were prepared with medium-viscosity alginate at 1% m/v and CMC at 1% m/v in distilled water. The biopolymers were solubilized in a magnetic stirrer at 750 rpm for 1 h at 25 °C. After achieving homogeneity, precross-linking began by slowly dripping a 1% m/v CaCl_2_·2H_2_O solution with a Pasteur pipet, maintaining stirring. A proportion of 7.5 mL of cross-linking solution was used for each gram of alginate present in the solution. Once the homogenization process was complete, the solutions were transferred to polystyrene Petri dishes (6 cm × 6 cm × 1 cm) in aliquots of 4.055 g (equivalent to 4.000 μL), and these were placed in BOD at a temperature of 40 °C and controlled ventilation for 20 h. After complete drying, each membrane was immersed in 10 mL of an aqueous solution of CaCl_2_·2H_2_O at 2.5% or 5.0% (m/v) for 15 min and subsequently washed in 10 mL of distilled water. The films were then placed on polystyrene plates placed again at BOD at room temperature for 48 h for final drying.

#### 2.2.3 Group Arrangement

For the characterization tests, the films were divided into six groups, three in 2.5% CaCl_2_ cross-linking and three in 5.0%, these conditions were selected based on prior optimization. The arrangement of the groups is shown in following table (Table 1):

**Table 1:** Experimental Groups of Alginate/CMC Films with Different CaCl_2_ Cross-Linking and ConA Treatments

group	cross-linking (CaCl_2_)	treatment
1	2.5%	control (NaCl 150 mM)
2	2.5%	ConA 50 μg/mL
3	2.5%	ConA 200 μg/mL
4	5.0%	control (NaCl 150 mM)
5	5.0%	ConA 50 μg/mL
6	5.0%	ConA 200 μg/mL

#### 2.2.4 Visual Appearance

The evaluation of the visual appearance of the films was recorded by digital photography using images captured with a digital camera (Nikon Coolpix L810 16.1 megapixels), which considered the homogeneity, continuity and cohesion of the film.

#### 2.2.5 Thickness of Films

The thickness of the films was measured on a digital micrometer in 10 random positions. Films with any visible defect were discarded, and then the arithmetic mean of these values was taken.

#### 2.2.6 Swelling

For this analysis, film samples measuring 6 cm × 1 cm were previously weighed and then exposed to 10 mL of distilled water for 24 h in a B.O.D incubator at 25 °C. After immersion, the samples were removed from contact with the fluid, excess moisture was blotted with filter paper if present, and finally reweighed. The degree of swelling (DS) was calculated based on the initial total mass of the sample according to eq 1, where Mu represents the mass of the wet sample and Mi is the initial mass of the sample.

1 DS=Mu−Mi

This equation provides a quantitative measure of the swelling extent experienced by the film samples during the experimental procedure.

#### 2.2.7 Mass Loss

The percentage of mass loss was determined by immersing them in 10 mL of distilled water for 24 h. After the exposure period, the samples were dried in BOD at 37 °C until they reached a constant mass. The mass loss (ML) was determined by the relationship between the final dry mass and the initial mass of the samples, according to eq 2, where Ms is the mass of the sample after drying and Mi is the initial mass of the sample.

2 ML=Mi−Ms

#### 2.2.8 Hemagglutinating Activity

The hemagglutinating activity test was carried out in a Petri dish (6 cm × 1 cm), with the films divided into six groups, three at 2.5% cross-linking and three at 5.0%. Samples of 2 mL of 25 mM Tris-HCl (pH 7.6) were added to all groups and then 3 mL of 3% native rabbit erythrocytes and subsequent incubation for 2 h, thus obtaining the result.

#### 2.2.9 Title of Hemagglutination Activity

In 5 mL test tubes, an aliquot of 50 μL of 25 mM Tris-HCl at pH 7.6 was added, followed by 50 μL of serial dilution, with the final tube reaching a total volume of 100 μL. This dilution was performed in each of the six groups of films, which were continuously extracted with PBS using a homogenizer. This process was repeated at 15, 30, 60, 0, 120, and 150 min. Subsequently, 50 μL of 3% native rabbit erythrocytes were added at all time points. After 3 h and 6 h incubation periods, monitoring ensued. Tests were performed in triplicate.

### 2.3 Physical Characterization

#### 2.3.1 Differential Scanning Calorimetry

The thermal properties of the dried film were measured on the differential scanning calorimetry (DSC) Q20 (TA Instruments). The samples were placed in hermetic aluminum cells and evaluated from 35 to 280 °C at a heating rate of 10 °C/min under a nitrogen atmosphere.

#### 2.3.2 FTIR Spectra

The FTIR spectra of the films were registered in FTIR spectrophotometer (PerkinElmer Spectrum Two), using the ATR (attenuated total reflectance) mode to provide information on the functional groups. The spectra were recorded between 400 cm^–1^ and 4000 cm^–1^ by 32 scans integrated per spectrum at a resolution of 4 cm^–1^.

#### 2.3.3 Scanning Electron Microscopy

The micrographs were taken using a scanning electron microscope (SEM) with a field emission gun, FEI brand, model Quanta FEG 250, with an accelerating voltage of 15 kV and spot 4.0 on the secondary electron and backscattered electron detectors. To take the micrographs, the samples were fixed to an aluminum substrate (stub) using double-sided carbon adhesive tape and grounded with carbon paint.

#### 2.3.4 Rugosity Profile

The quantitative analysis of average surface roughness (Ra) and topography of the films were measured by confocal laser scanning microscope (CLSM; LSM700, Carl Zeiss). The roughness parameters were determined by the visualization software using a surface area of 100 μm^2^.

### 2.4 Angiogenic Activity Assessment

#### 2.4.1 Chick Embryo Chorioallantoic Membrane Assay

The angiogenic effect of ConA film was assessed by the chorioallantoic membrane (CAM) assay, based on the protocol described by Auerbach et al. with modifications.^22^ Thirty-five fertilized hen eggs (*Gallus gallus domesticus*) were housed in a BOD chamber (model SL224) with a humidified atmosphere (70 ± 4% relative humidity) and controlled temperature (35 °C). On the seventh incubation day, the eggshell was subjected to a circular opening at the egg-wider base to remove the shell membrane. Then, the eggs were sealed and reincubated. On the 12th incubation day, the eggs were randomized (five eggs/group) into seven treatment groups: (1) negative control film 2.5% cross-linking; (2) negative control film 5% cross-linking; (3) 50 μg/mL ConA film 2.5% cross-linking; (4) 50 μg/mL ConA film 5% cross-linking; (5) 200 μg/mL ConA film 2.5% cross-linking; (6) 200 μg/mL ConA film 5% cross-linking; and (7) 20 μL healing oil Dersani (angiogenesis inducer).

After 72 h, the CAMs were fixed in methanal (CH_2_O; 10%), and the angiogenic effect was quantified from captured images (Nikon Coolpix L810 16.1 megapixels). The vascularization percentage was determined by ImageJ (version 1.51 j8) software. The length and caliber of blood vessels, number of complexes and junctions were determined by AngioQuant (version 1.33) software.

Histological analysis of CAM was performed after tissue processing and hematoxylin–eosin (HE) staining of samples. The histological parameters (neovascularization, presence of inflammatory elements, fibroblasts, and thickening of the chorioallantoic membrane) were visually classified according to the quantity and/or intensity in the CAM histological sections using a light microscope (Olympus, model BH2) with a 40× objective lens. The data obtained were transformed into quantitative variables, assigning the following scores: (0) absent, (1) discrete, (2) moderate, and (3) intense.

#### 2.4.2 Immunohistochemistry of CAM

For immunodetection of angiogenic factors in treated CAMs, methanal-fixed and paraffin-embedded samples were cut at 4 μm on silane-coated (4% SiH_4_) microscopic slides. Dewaxed samples underwent heat-induced antigen retrieval in a water bath (97 °C) immersed in citrate buffer (10 mM; pH 6.0) for 40 min, followed by quenching of endogenous peroxidase activity with hydrogen peroxide (3% H_2_O_2_) for 20 min. Subsequently, each histological sample was incubated with primary antibodies against vascular endothelial growth factor (VEGF; 1:400 mouse monoclonal IgG, sc-53462; Santa Cruz Biotechnology) or transforming growth factor beta (TGF-β; 1:400 polyclonal rabbit IgG, sc-7892; Santa Cruz Biotechnology) in a humid chamber at 4 °C overnight. The immune complexes were treated with peroxidase-conjugated affinipure goat secondary antibody (1:500 antimouse IgG, 113-035-003; Jackson ImmunoResearch Laboratories) for 3 h at room temperature. The immunoreactivity was detected by Novocastra DAB (1:50) chromogen substrate for 10 min, and the sections were counterstained with Harris’ hematoxylin.^23^

To quantitatively evaluate the VEGF and TGF-β levels, an average percentage of positive cells was determined derived from 5 photomicrographs of random areas obtained in a light microscope with magnification of 40× by ImageJ (version 1.5 j8) software.

### 2.5 Statistics Analysis

The data analysis was carried out using the GraphPad Prism 6.0 statistical program. Data were analyzed with two-way ANOVA, using the geometric mean of the triplicates and the standard deviation of the mean as central data. Significant differences between means were identified using the Tukey test. For macroscopic, histological, and immunohistochemical evaluations regarding angiogenic analysis, the data obtained were analyzed by one-way ANOVA and Tukey’s post hoc test using the SigmaStat software (version 3.5). For all data, *p* < 0.05 was considered significant.

## 3 Results and Discussion

### 3.1 Film Characterization

#### 3.1.1 Visual Aspect

This study offers a new perspective on the therapeutic application of plant lectins by incorporating these proteins into sodium alginate and carboxymethylcellulose films, using them as vehicles for compounds with angiogenic properties. Lectins in alginate films have already been studied regarding their antimicrobial activity against candidiasis, *Staphylococcus aureus* and *Pseudomonas aeruginosa*.^16,24^ Characterizing these films is crucial because their properties can significantly influence the outcomes.

Regarding the visual characterization of the alginate/CMC polymeric films produced in this study, we observed that they are macroscopically homogeneous and free of cracks (Figure 1A–F). Additionally, these films are transparent, flexible, have smooth surfaces, and contain small air bubbles. Small transparency contrasts are observed in films with higher cross-linking (Figure 1D,F) and higher amounts of ConA lectin (Figure 1C,F) compared to the control (Figure 1A,D).

**Figure 1:**
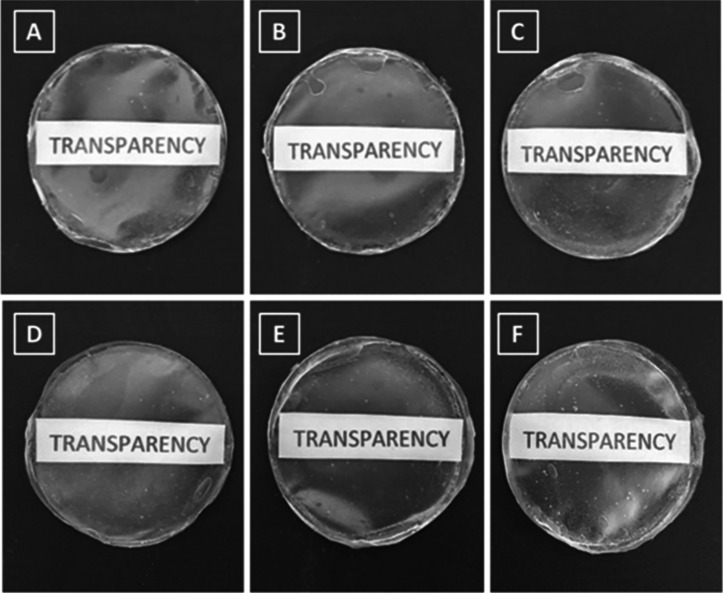
Visual appearance of cross-linked alginate/CMC films with or without ConA. (A) Control: alginate/CMC film at 2.5% cross-linking containing NaCl; (B) ConA 50 μg in 2.5% cross-linking; (C) ConA 200 μg at 2.5% cross-linking; (D) control at 5% cross-linking; (E) ConA 50 μg in 5% cross-linking; (F) ConA 200 μg in 5% cross-linking.

More transparent films tend to be more effective, providing better monitoring of macroscopic aspects of the study target, such as wounds,^25^ due to greater homogeneity and better dispersion of components. Macedo et al.^16^ observed that films containing the ConA and the antibiotic Gentamicin were also more transparent when compared to the control. The same was observed by Bazán et al.^24^ by including the lectins ConBr and MaL in alginate films. Transparency results from the effective dilution of the compounds in the films, indicating that alginate can dilute and diffuse proteins.^26^ In our tests it is possible to observe a high level of transparency, similar in all groups, indicating a complete and uniform dilution of the compounds present. For an effective film, it must present certain characteristics, such as good resistance, adhesion, and mechanical support in the tissues to which it will adhere, among others.^27^

Regarding the thickness of the alginate/CMC films, as shown in Table 2, there are no statistically significant differences between the treated groups and the control group. These results suggest that variations in cross-linking conditions and the presence or absence of ConA have no impact on film thickness.

**Table 2:** Film Thickness (ConA) of Alginate and CMC (1:1) Obtained with Cross-Link Concentrations of 2.5% and 5%^a^

cross-linking (2.5%)	thickness (μm)
group 1	0.0355
group 2	0.038
group 3	0.0355

cross-linking (5.0%)	thickness (μm)
group 1	0.0355
group 2	0.038
group 3	0.0375

a Mean ± standard deviation. Not significant (*p* > 0.05) Tukey test.

The results highlighted in Table 3 regarding swelling and mass loss reveal significant statistical differences within the same cross-linking groups and between different cross-linking agents. It is evident that the films containing ConA at 2.5% cross-linking exhibited a higher swelling rate compared to the control film. At 5% cross-linking, however, the swelling degree decreased for all groups, with group 2 showing the highest value among the ConA-containing films.

**Table 3:** Degree of Swelling and Mass Loss of Alginate and CMC (ConA) Film (1:1) Obtained with Crosslinking Concentrations of 2.5% and 5%^a^

cross-linking concentration (2.5%)	degree of swelling (mg H_2_O film)	mass loss
group 1	596.4 ± 0.3^Aa^	23.5 ± 0.25^aA^
group 2	675.6 ± 1.47^Ba^	20.0 ± 0.26^bB^
group 3	664.0 ± 0.87^Ca^	20.3 ± 0.37^bB^

cross-linking concentration (5.0%)	degree of swelling (mg H_2_O film)	mass loss
group 1	584.4 ± 0.75^aB^	22.1 ± 0.34^aB^
group 2	609.4 ± 0.60^bB^	21.3 ± 0.46^aB^
group 3	572.0 ± 1.41^cB^	19.8 ± 0.20^bB^

a Mean ± standard deviation. Lowercase letters represent statistical differences within the same cross-link agent, and uppercase letters represent statistical differences between cross-link agents (*p* < 0.05), Tukey test.

Analysis of mass loss showed that at 2.5% cross-linking, control films had the greatest loss, while at 5% cross-linking, films with the highest concentration of ConA had the lowest mass loss. This indicates that increased cross-linking implies lower swelling capacity, since cross-linking prevents water from penetrating the interior of the film, and consequently less mass loss due to the outermost groups, which must be related to mass loss, being more protected after cross-linking. Among films prepared with varying concentrations of ConA using CaCl_2_ as a cross-linking agent, control films with 2.5% show statistically significant differences compared to all other groups. These findings underline the significant influence of ConA addition and cross-linking on the swelling and mass-loss properties of alginate/CMC films.

According to Boateng et al.,^28^ the ideal thickness, however, will provide high absorption capacity, and the texture and porosity of the material also controls this property. The thickness of the film is a key factor for success. Very thin films are more fragile, adhere less, have limited mechanical properties, and dissolve easily when applied to tissue. However, the thickness primarily relates to the film’s intended purpose.^29^ The literature does not indicate an ideal thickness for polymeric films, since this property depends on the body region to be treated, which shows that the thicknesses of the films obtained in this work have potential for application in wound treatment.

#### 3.1.2 Hemagglutinating Activity and Title of Hemagglutinating Activity

Another crucial characteristic of these films is their absorption capacity. Wounds on the surface of the skin can generate exudate, and when in excess, they hinder the adequate transport of oxygen and water, in addition to promoting the emergence of edema and bacterial infections.^30^ For this reason, films must promote appropriate water absorption to prevent the accumulation of exudate and thus promote healing. Furthermore, a film with good water retention maintains wound moisture, being crucial for adhesion, differentiation, cell proliferation, pain reduction and activation of collagen synthesis.^31,32^

The analysis of hemagglutinating activity in the films showed the expected absence of activity in the control films (group 1) (Figure 2A,D) and the presence of activity in the ConA-containing groups (groups 2 and 3) (Figure 2B,C,E,F). An additional finding highlights the influence of cross-linking on ConA-containing alginate/CMC films, where activity was more prominent in 2.5% cross-linked alginate/CMC films (Figure 2B,C) compared to 5% cross-linked alginate/CMC films (Figure 2E,F). Although no previous studies directly address ConA release from alginate/CMC films, the trend is consistent with fundamental principles of polymer cross-linking and diffusion.^15^

**Figure 2:**
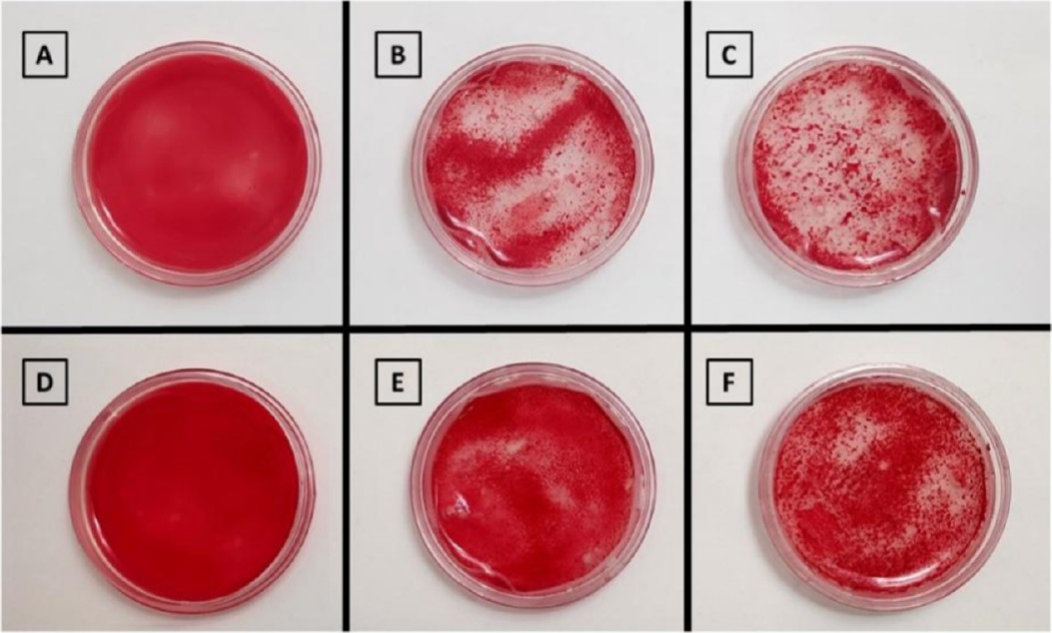
Assessment of the hemagglutinating activity of films CMC/alginate (1:1) containing (A) 2.5% cross-linking control; (B) 2.5% cross-linking ConA 50 μg; (C) 2.5% cross-linking ConA 200 μg (D) 5% cross-linking control; (E) 5% cross-linking ConA 50 μg in (F) 5% cross-linking ConA 200 μg.

To confirm the variation in lectin release rates in films with different degrees of cross-linking (2.5% and 5%), hemagglutinating activity titers were evaluated. As shown in Figure 3, films with 200 μg of ConA and 2.5% cross-linking reached maximum release in 90 min and remained stable until 150 min, with hemagglutinating activity of 8 HU. In contrast, films with the same concentration but 5% cross-linking reached maximum release in 120 min and remained stable until 150 min, with an activity of 16 HU.

**Figure 3:**
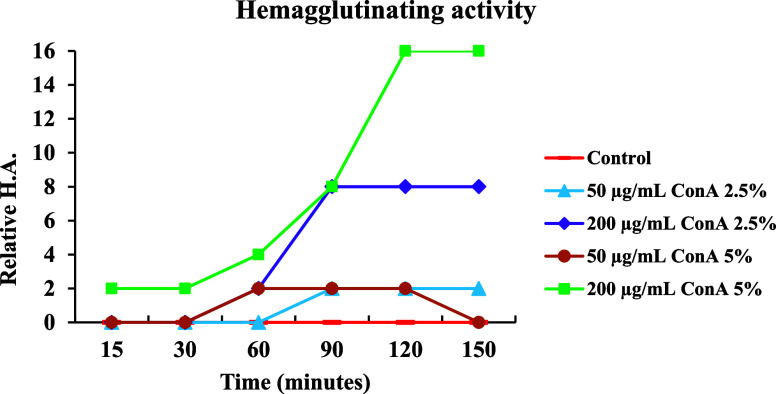
Film hemagglutinating activity (ConA) titers after the two types of cross-linking processes.

For films with 50 μg of ConA, maximum release occurred in 60 min for 2.5% cross-linking and in 90 min for 5% cross-linking. Notably, activity was lost after 150 min in the 5% cross-linked films.

Alginate-based films tend to have good water absorption, as this polymer acts as an antidehydration agent, being recommended for dressings on wounds with a high exudate content.^33,34^ In this work, we found that the films in all groups had a good water absorption capacity, as indicated by the degree of swelling shown in Table 3, proving to be physically stable in the presence of liquid media, maintaining the absorption capacity that is necessary for applications such as dressings. These data are corroborated by Gontijo and Bierhalz,^35^ who found similar data in their tests with alginate and carboxymethylcellulose (CMC) films incorporated with sodium diclofenac.

### 3.2 Physical Characterization

#### 3.2.1 Differential Scanning Calorimetry Analyzes

The thermal processes of combined/or modified films and its pure counterpart were assessed by differential scanning calorimetry analysis (DSC). The results are shown in Figure 4 and compared to the thermal transitions of pristine and combined alginate films, exhibiting the influence of the modifying moiety on the anionic linear polysaccharide stability. The DSC thermograms of pure alginate membrane with 2.5% of CaCl_2_ exhibited an endothermic peak at 92.40 °C related to the evaporation of absorbed water or by vaporization of volatile components for both content of CaCl_2_ (Figure 4A, group I—black curve). For the pure alginate membrane with 5% of CaCl_2_ (Figure 4A—yellow curve) the endothermic peak shifted to lower temperature (84.50 °C).^36^

**Figure 4:**
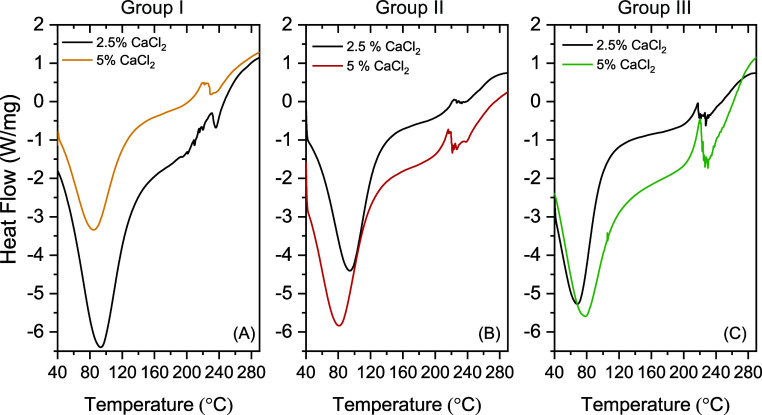
DSC thermograms. (A) Group I: alginate/CMC film with NaCl and 2.5% or 5% of CaCl_2_. (B) Group II: alginate/CMC film with ConA and 2.5% or 5% of CaCl_2_. (C) Group III: alginate/CMC film with NaCl and ConA and 2.5% or 5% of CaCl_2_.

In the case of the addition of ConA lectin to the alginate/CMC films, the DSC curves showed an endothermic peak at 94 °C (for 2.5% CaCl_2_) and 81 °C (for 5% CaCl_2_) followed by a small exothermic peak (Figure 4B, group II, black and red curves, respectively). By analyzing the exothermic point in the temperature range 220–230 °C for both reported samples, we noticed that from there, partial depolymerization of the alginate chain begins. As for alginate/CMC films with ConA and NaCl, the endothermic peak changed to 69 °C (for 2.5% CaCl_2_) and 78 °C (for 5% CaCl_2_) and the endothermic region changed to a higher temperature range (Figure 4C, group III, black and green curves, respectively). Alginate/CMC films containing ConA and NaCl tend to retain less water, which volatilizes more easily compared to pure alginate/CMC films.

These findings, when correlated with the water affinity, indicate that the presence of the lectin made the membrane-water interaction less intense, leading to a shift in the first endothermic peak when compared to the pure sample. Furthermore, the presence of Con A and NaCl in the composition did not change the stabilizing capacity of the membrane, since the thermal behaviors at high temperature were similar, but significantly changed the temperature of the dry polymer glass transition (related to the endothermic peak).^36^

Regarding aspects of physical analysis, partial depolymerization of the alginate chain was observed when the exothermic point reached the reported samples in a temperature range of 220–230 °C.^37^ Furthermore, films cross-linked with ConA and NaCl tend to retain less water, which in turn volatilizes more easily than in the control film. Such findings, when correlated with water affinity, indicate that the presence of the lectin made the film water interaction less intense, leading to a shift in the first endothermic peak when compared to the pure sample. Furthermore, the presence of ConA and NaCl in the composition did not change the stabilizing capacity of the membrane, as the thermal behaviors at high temperatures were similar, but it significantly changed the glass transition temperature of the dry polymer (related to the endothermic peak).^36^

#### 3.2.2 FTIR Analyzes

The FTIR spectra of groups I (with NaCl), II (with ConA) and III (with NaCl and ConA) were recorded and compared (Figure 5). Initially, the spectra of the alginate/CMC film (group I), cross-linked with Ca^2+^ under both CaCl_2_ conditions, exhibited significant absorption bands related to hydroxyl and carboxylic functional groups (Figure 5, group I, curves a and b). In the range of 3220 cm^–1^ to 3350 cm^–1^, the intermolecular/intramolecular hydrogen-bonded O–H stretching vibration of alginate/CMC film was prominently observed, overlapping the structural N–H vibration.^38,39^ Distinct stretching vibrations of C–H were noted at 2880 cm^–1^ and 2935 cm^–1^. The bands at 1560 cm^–1^ and 1411 cm^–1^ were assigned to asymmetric and symmetric stretching vibrations of COO^–^ groups. At lower frequencies, the bands at 1034 cm^–1^ and 924 cm^–1^ were attributed to C–O stretching, incorporating contributions from C–C–H and C–O–H deformation, as well as absorption band of α-(1,4) glycoside bond, respectively.^40^ The pronounced absorption band at 1034 cm^–1^ is associated with glycosidic linkages in both CMC and alginate.

**Figure 5:**
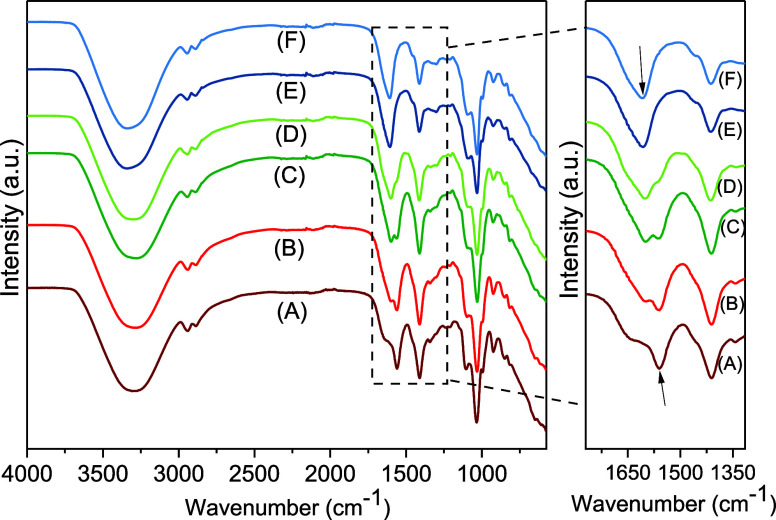
FTIR analyzes. Group I: (A) spectra of alginate/CMC film with NaCl and 2.5% CaCl_2_, and (B) 5% of CaCl_2_. Group II: (C) spectra of alginate/CMC film with lectin ConA with 2.5%, and (D) 5% of CaCl_2_. Group III: (E) spectra alginate/CMC film with NaCl and lectin ConA with 2.5%, and (F) 5% of CaCl_2_.

In both conditions of alginate/CMC films with ConA lectin (Figure 5, groups II and III, curves C–F), the characteristic bands of the pure sample were evident in the control sample, indicating the preservation of structure and primary interactions. The addition of ConA to the film composition resulted in changes in the region between 1750 cm^–1^ and 1480 cm^–1^, indicating a possible interaction between the active groups of ConA and the functional groups of the film, especially the carboxylate groups (COO^–^). These FTIR data suggest that ConA is anchored in the alginate matrix not only due to the cross-linked nature of the film chain, but also through possible direct interactions or cross-linking with these functional groups. FTIR data suggest that ConA is anchored in the alginate matrix due to the cross-linked nature of the film chain, with minimal chemical interactions (or chemical bonds).^40^ It is noteworthy that the presence of ConA gradually suppressed the contribution at 1560 cm^–1^ and revealed a band at 1607 cm^–1^, potentially attributed to a cross-linked lectin–alginate network formed by carbonyl groups (Figure 5, representative arrows).^41^ Consequently, the synergistic chemical environment between the film and the lectin facilitated the complete action of ConA as a hemagglutinating agent.

Approaches to studying the molecular interactions of the film samples and their components revealed bands attributable to the stretching (axial deformation) of the intertwined hydroxyl structural groups (−OH and –OH), characteristic of natural polysaccharides.^27^ In relation to those found in regions of low-frequency bands of Ca–C stretching vibrations, the rotational vibrations of the CH_3_ group are attributed to different skeletal vibrations.^36–43^ Nevertheless, the results show a clear interplay between the lectin and the chemical groups of the film as shown in Table S1.^44^

#### 3.2.3 Scanning Electronic Microscopy

The morphological characterization by scanning electron microscopy (SEM) of films composed of alginate and carboxymethyl cellulose (CMC) incorporated with *C. ensiformis* lectin (ConA) revealed images that evidenced distinct but complementary aspects of the morphology of the films. The magnitude presented is 5000× (20 μm) as illustrated in Figure 6.

**Figure 6:**
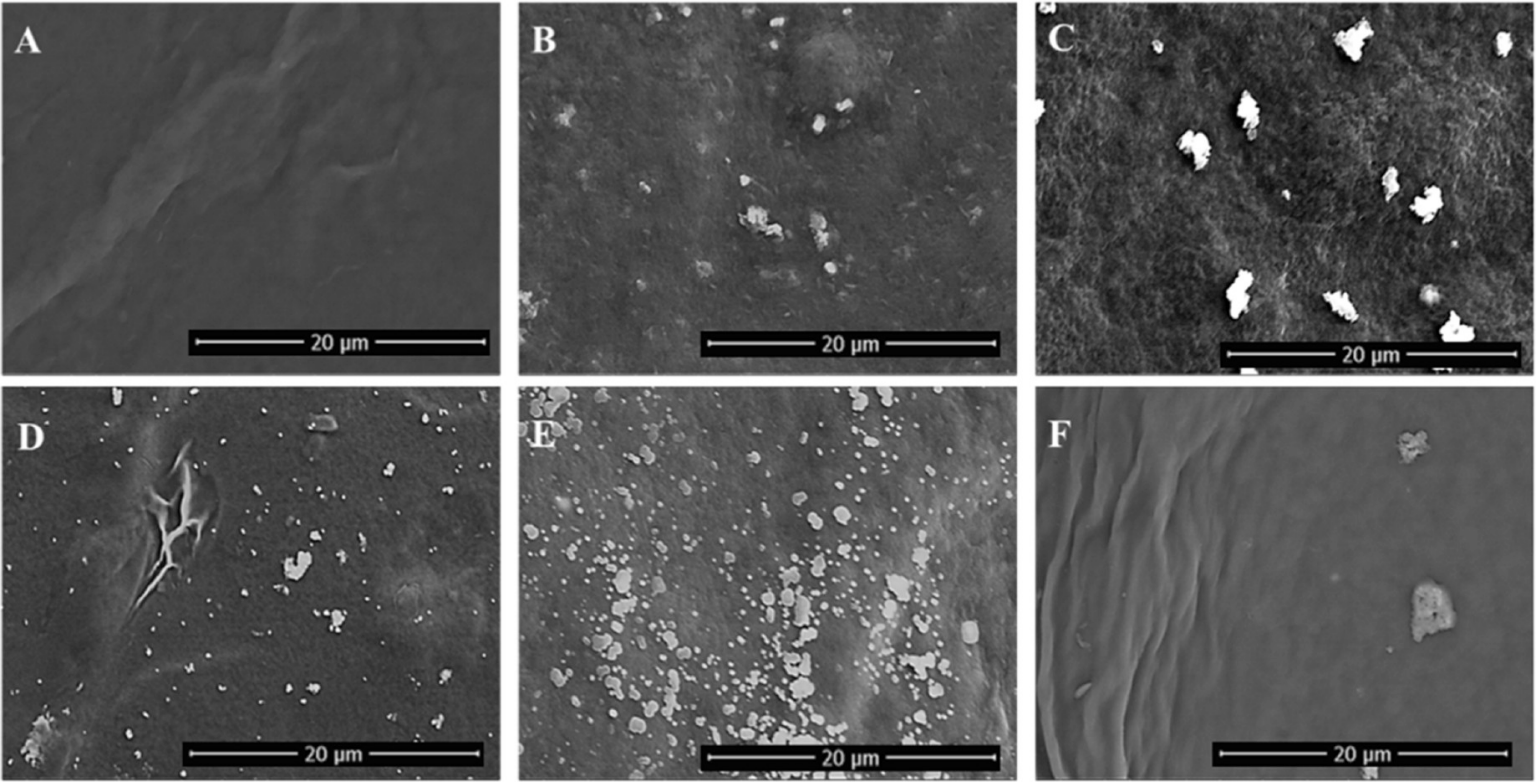
Scanning electron microscopy of the films: ConA (50 μg/mL) with 2.5% (A); ConA (50 μg/mL) with 5.0% (B); ConA (200 μg/mL) with 2.5% (C); ConA (200 μg/mL) with 5.0% (D); control group with 2.5% (E); control group with 5.0% (F).

The micrographs highlight a relatively rough (Figure 6A–C,F) surface with interconnected (Figure 6D) regions that favor cell adhesion and proliferation. These morphologies are compatible with ideal substrates for supporting endothelial cells, promoting migration and the formation of capillary structures.^45^ A marked contrast is observed in certain areas, which may indicate regions with higher ConA density (Figure 6C). The presence of this lectin is crucial, as it has a high affinity for mannose and glucose residues in cell surface glycoproteins, potentially facilitating cell-material interactions.^26^ ConA is known to induce bioactive responses such as cell proliferation and modulation of the inflammatory response, as well as to enhance tissue revascularization. Surface roughness also promotes the formation of new blood vessels, providing a permissive interface for in situ revascularization.^46^ The morphological characterization presented here strengthens the argument that these biomaterials may serve as bioactive platforms to support angiogenesis.

#### 3.2.4 Roughness Profile of the Films

The dynamics of surface roughness of groups I (with NaCl), II (with ConA) and III (with NaCl and ConA) were examined by confocal laser scanning microscopy. For each sample, the average roughness (Ra) and surface topography were displayed in Figure 7. The Ra values of the alginate/CMC films with NaCl and 2.5% or 5% CaCl_2_ (group I), alginate/CMC films with ConA lectin with 2.5% or 5% CaCl_2_ (group II) and alginate/CMC films with NaCl and ConA lectin with 2.5% or 5% CaCl_2_ (group III) were 0.480 ± 0.08 μm and 1.430 ± 0.06 μm; 0.540 ± 0.04 μm and 3.370 ± 0.04 μm; 0.910 ± 0.07 μm and 4.110 ± 0.09 μm, respectively. A significant increase was observed in surface roughness profile in the presence of ConA and NaCl. Also was observed that the surface roughness of each pair increased with increasing CaCl_2_ concentration and consequently, more surface area was available for lectin attachment through nonspecific interactions. It was easily noticed that the surface roughness substantially increased with increasing the ConA content and in the presence of NaCl, regardless of the CaCl_2_ amount compared to the unmodified membrane. This behavior is explained as the addition of ConA in the film might result in the formation of an amorphous-like structure and the creation of more structural pores and sorption centers due to the large difference in the miscibility degree between the film and the ConA lectin, which produced significant differences in surface roughness. A film with greater surface roughness could provide a more effective surface for reactions that occurs exclusively in that interface.^47^

**Figure 7:**
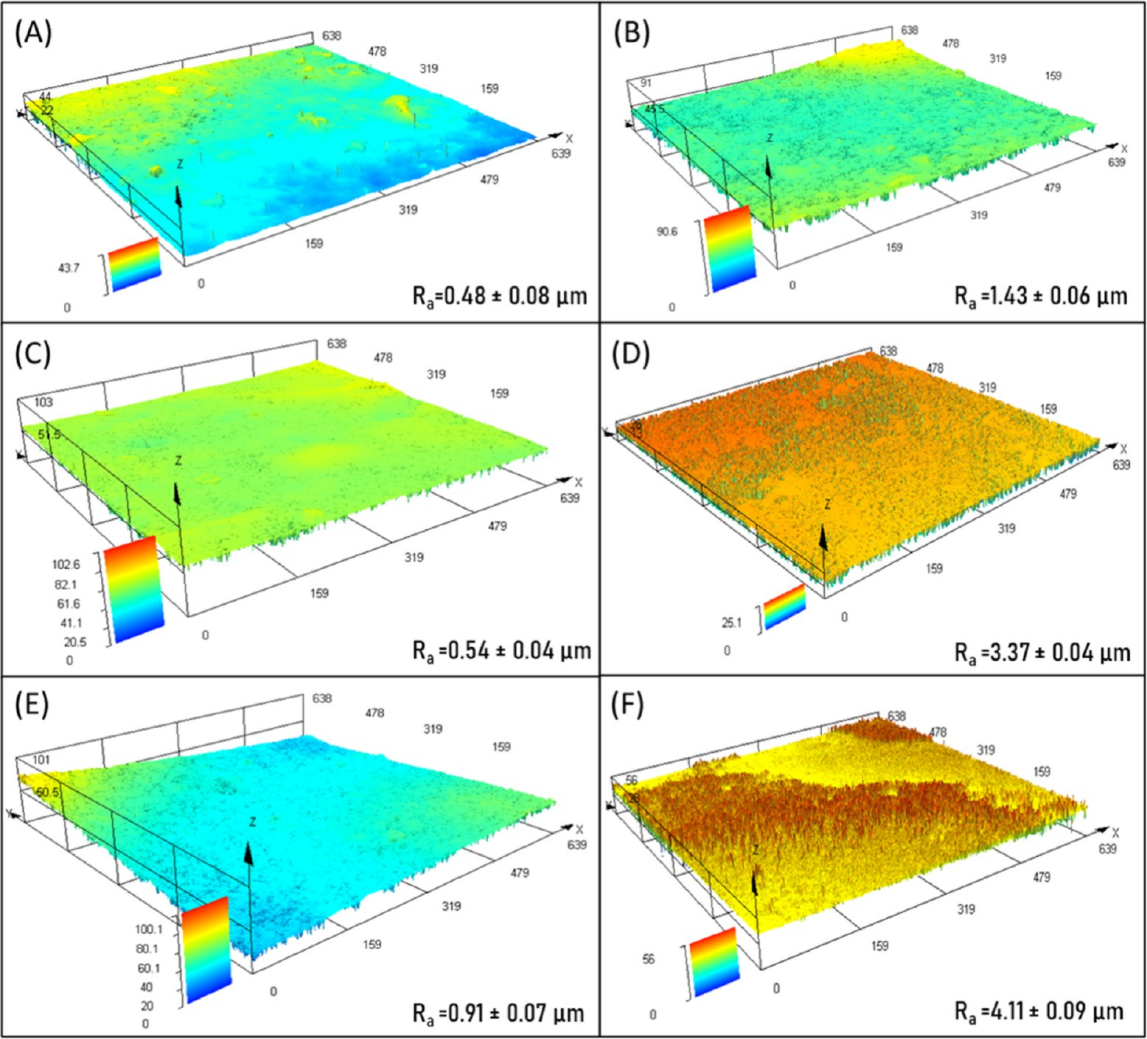
Confocal laser scanning microscopy images showing surface roughness (Ra) of the films: (A,B) alginate/CMC films with NaCl and 2.5% or 5% CaCl_2_, respectively; (C,D) alginate/CMC films with ConA and 2.5% or 5% CaCl_2_, respectively; (E,F) alginate/CMC films with NaCl and ConA and 2.5% or 5% CaCl_2_, respectively.

It was seen that the surface roughness of the films increased with increasing CaCl_2_ concentration and, consequently, more surface area was available for lectin binding through nonspecific interactions. It was easily noticed that the surface roughness increased substantially with increasing ConA content and in the presence of NaCl, regardless of the amount of CaCl_2_ compared to the unmodified film. This behavior is explained because the addition of ConA into the matrix can result in the formation of an amorphous structure and the creation of more structural pores and sorption centers due to the large difference in the degree of miscibility between the film and the lectin, which produced significant differences in the surface roughness. A film with greater surface roughness could provide a more effective surface for reactions that occur exclusively at this interface.^26^ The roughness of dermal dressings influences cell adhesion and proliferation, as well as the shape taken on by cells when cultured on a given surface. As such, roughness is an important factor that determines the biocompatibility of a material.^48^ According to Milleret et al.,^49^ the rougher the surface, the greater the adhesion of platelets and the formation of thrombin, which are favorable conditions for accelerating the healing of skin lesions. It has also been reported that the greater the roughness of a skin dressing, the greater the anchorage between the surface and the necrotic tissue of the wound.^50^

### 3.3 Angiogenic Activity

#### 3.3.1 ConA Angiogenic Activity in CAM Assay

The CAM assay results demonstrated that ConA induced a pronounced angiogenic effect, since it promoted a significant increase in all the parameters analyzed, including percentage of vascularization (Figure 8), length and caliber of blood vessels, and number of complexes and junctions (Table 4) at 50 and 200 μg/mL concentrations (2.5 or 5% cross-linking) when compared to the negative control (*p* < 0.05).

**Figure 8:**
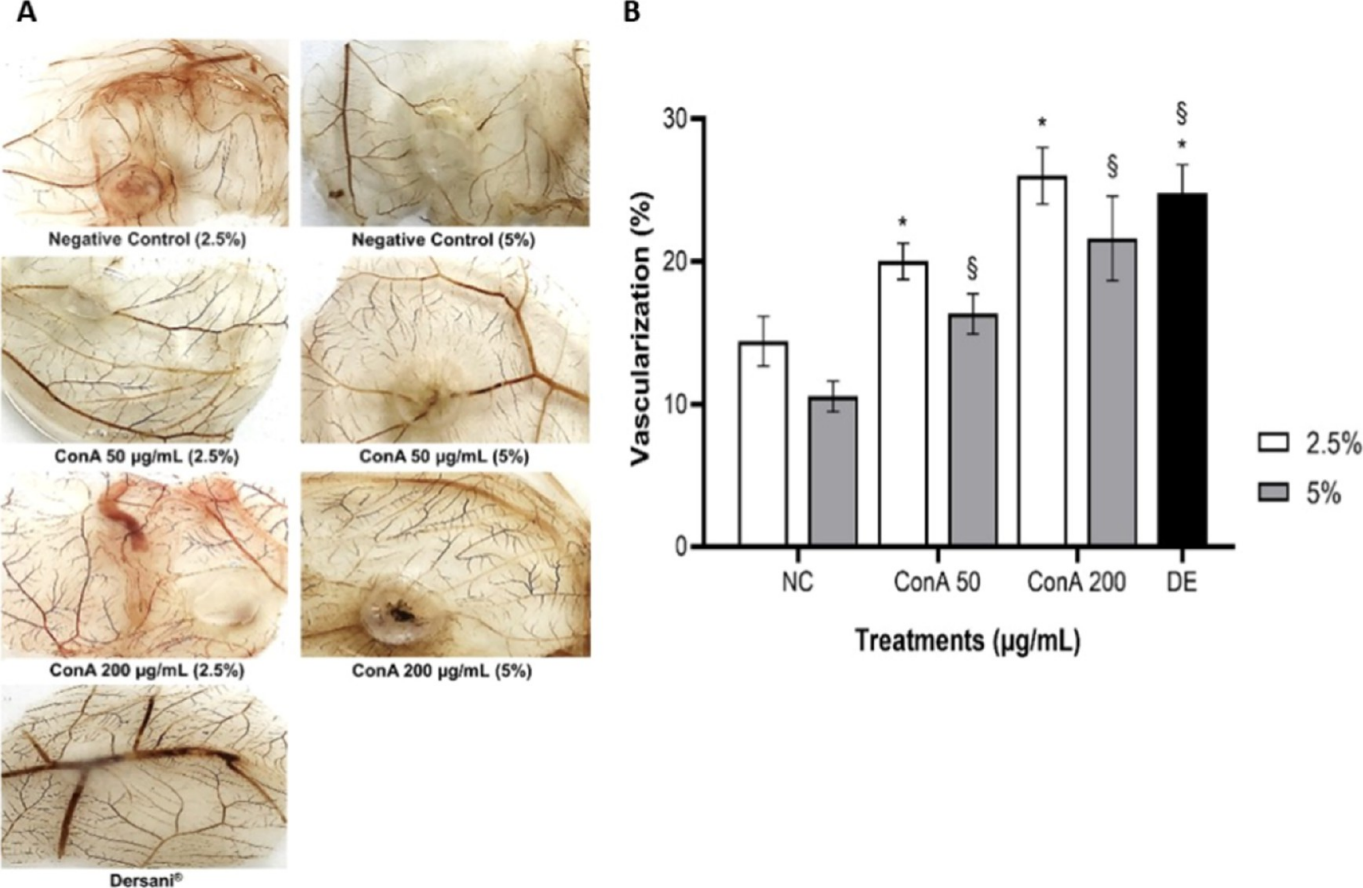
Angiogenic effect of ConA by chick embryo chorioallantoic membrane (CAM) assay. (A) Representative images of different CAM after 72 h of treatments. (B) Average vascularization percentage obtained from each treatment by ImageJ software. NC: negative control: cross-linking solution + 150 mM NaCl; ConA: cross-linking solution + concanavalin A lectin (50 or 200 μg/mL) + 150 mM NaCl. DE: healing oil Dersani (angiogenesis inducer). ANOVA and posthoc Tukey test. * Significant difference compared to the negative control 2.5% (*p* < 0.05); § significant difference compared to the negative control 5% (*p* < 0.05).

**Table 4:** Means ± Standard Deviation of Parameters Analyzed in Chick Embryo Chorioallantoic Membranes (CAM), Treated with Different Concentrations of ConA by AngioQuant Software^a^

treatments (μg/mL)	length (pixel)	caliber (pixel)	number of complexes	number of junctions
Dersani	343.1 ± 23.2*	2155.0 ± 217.7*	267.0 ± 13.7*	299.2 ± 23.3*
2.5%				
negative control	115.2 ± 28.0	650.9 ± 286.3	98.6 ± 41.1	55.8 ± 28.7
ConA 50	200.5 ± 50.0*	1420.0 ± 404.9*	127.2 ± 16.5*	116.5 ± 29.8*
ConA 200	299.7 ± 30.3*	2075.1 ± 394.6*	234.7 ± 14.5*	275.0 ± 20.3*
5%				
negative control	81.2 ± 12.2	677.5 ± 135.9	77.4 ± 13.0	40.2 ± 10.0
ConA 50	162.7 ± 34.0*	1590.0 ± 383.3*	98.8 ± 27.0*	105.5 ± 53.8*
ConA 200	202.8 ± 15.3*	1893.5 ± 352.7*	109.5 ± 17.2*	114.3 ± 20.8*

a Healing oil Dersani (angiogenesis inducer); negative control: cross-linking solution + 150 mM NaCl; ConA: cross-linking solution + concanavalin A lectin (50 or 200 μg/mL) + 150 mM NaCl. ANOVA and posthoc Tukey test. * Significant difference compared to the negative control (*p* < 0.05).

This effect was confirmed in the CAM histological analysis (Figure 9 and Table 5). In the ConA treated films, there was a significant increase in all histological parameters analyzed (neovascularization, presence of inflammatory and fibroblast cells and CAM thickening) at 50 and 200 μg/mL concentrations (2.5 or 5% cross-linking), showing its inducing effect of the angiogenic process.

**Figure 9:**
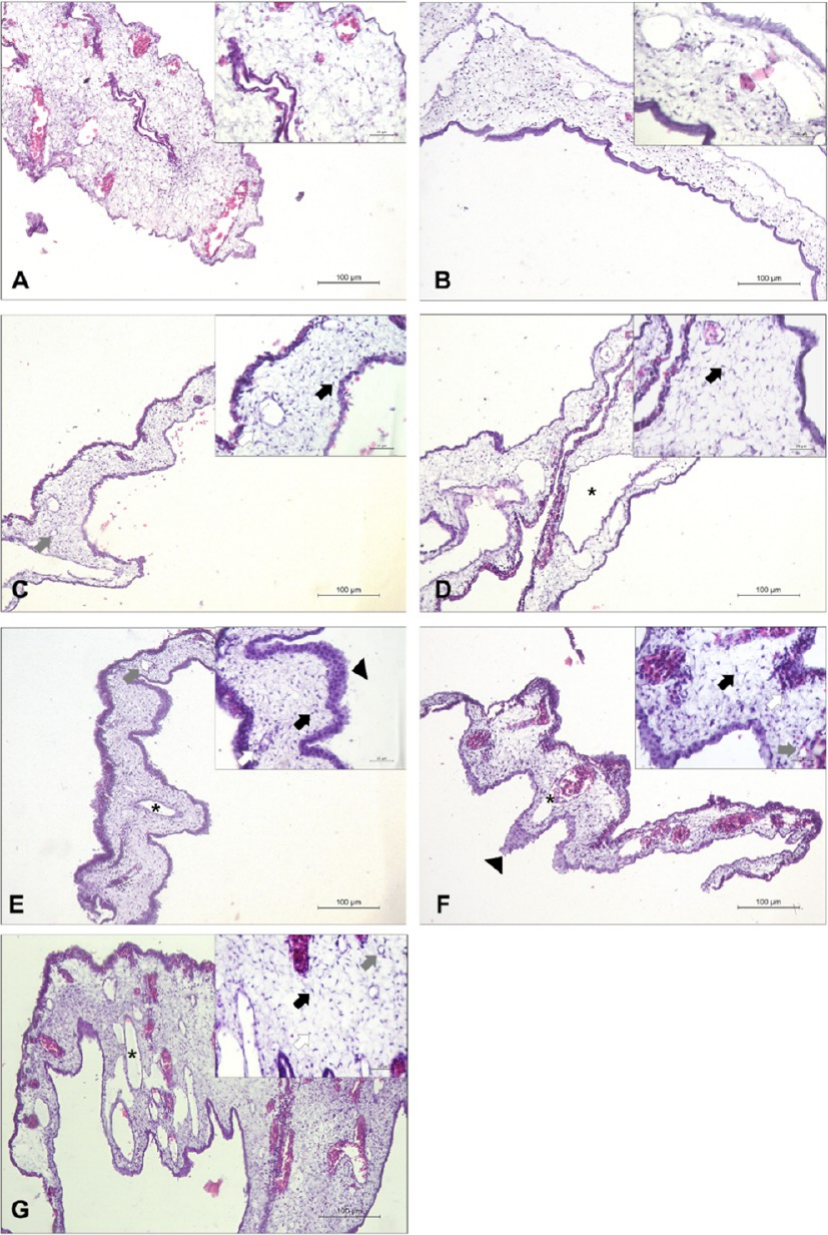
Representative photomicrograph of chick embryo chorioallantoic membranes stained by hematoxylin–eosin (HE), obtained from different treatment groups. (A) Negative control 2.5%; (B) negative control 5%; (C) ConA 50 μg/mL 2.5%; (D) ConA 50 μg/mL 5%; (E) ConA 200 μg/mL 2.5%; (F) ConA 200 μg/mL 5%. (G) Healing oil Dersani (angiogenesis inducer). Black arrow: fibroblasts; gray arrow: new vessels; white arrow: inflammatory cells; asterisk: pre-existing vessels; arrowhead: CAM thickening.

**Table 5:** Histological Analysis of Chick Embryo Chorioallantoic Membranes (CAM)^a^ ^,^ ^b^

treatments (μg/mL)	neovascularization	presence of inflammatory cells	presence of fibroblasts	thickening in chorioallantoic membrane
Dersani	2.5 ± 0.50*	2.4 ± 0.20*	2.5 ± 0.50*	2.6 ± 0.40*
2.5%				
negative control	1.0 ± 0.50	0.8 ± 0.20	1.0 ± 0.30	0.5 ± 0.20
ConA 50	1.7 ± 0.50*	1.5 ± 0.50*	1.8 ± 0.25*	1.3 ± 0.40*
ConA 200	2.8 ± 0.25*	2.5 ± 0.44*	2.8 ± 0.20*	2.0 ± 0.00*
5%				
negative control	0.5 ± 0.30	0.5 ± 0.20	0.5 ± 0.15	0.8 ± 0.20
ConA 50	1.2 ± 0.10*	1.2 ± 0.20*	1.6 ± 0.40*	1.0 ± 0.00
ConA 200	2.0 ± 0.33*	1.8 ± 0.22*	2.2 ± 0.15*	1.6 ± 0.20*

a Five CAM per treatment group were considered for the histological parameters analysis. Healing oil Dersani (angiogenesis inducer); negative control: cross-linking solution + 150 mM NaCl; ConA: cross-linking solution + concanavalin A lectin (50 or 200 μg/mL) + 150 mM NaCl. ANOVA and posthoc Tukey test. * Significant difference compared to the negative control (*p* < 0.05).

b Means ± standard deviation of histological parameters classified at a scale of 0–3.

Chronic inflammation, microbial infections, disturbance of angiogenesis, and reduced revascularization are some of the factors that hinder the wound healing process.^35,50^ The angiogenic and proliferative potential of the ConA lectin has been previously described through the Akt/ERK/Cyclin D1 axis.^19^ To date, there has been no study demonstrating the activity of conjugated lectin in films or in CAM analyses.

Our study found that incorporating ConA lectin into alginate and carboxymethylcellulose films did not compromise its angiogenic activity, indicating that the polymers’ presence did not affect the lectin’s angiogenic potential. The angiogenic effect is demonstrated by the increase in revascularization, length, and caliber of vessels and by the number of complexes and junctions. Findings like those of Rehman et al.,^31^ who observed the angiogenic effect of a hydrogel dressing incorporated with graphene oxide. Increased vessel thickness is a sign of the maturation of nascent blood vessels and is possibly a result of higher angiogenesis.

Other studies with the presence of alginate in dressings show the angiogenic effect, as is the case of Azarpira et al.,^51^ who demonstrated the potential role of VEGF-loaded alginate oxide particles in acellular collagen-alginate composite hydrogel to improve angiogenic activity. Silk fibroin/sodium alginate films promote revascularization and, consequently, angiogenesis.^52^

#### 3.3.2 ConA Modulates VEGF and TGF-β Expression in CAM

Immunohistochemistry of ConA treated CAM showed a significant increase in the expression of angiogenic factors analyzed in all treatment groups (Figure 10) compared to the negative control. Quantitative immunostaining analysis indicates that ConA-treated samples had significantly higher levels of TGF-β (Figure 10A) and VEGF (Figure 10B) expression when compared to the negative control (*p* < 0.05) at 50 and 200 μg/mL concentrations (2.5 or 5% cross-linking).

**Figure 10:**
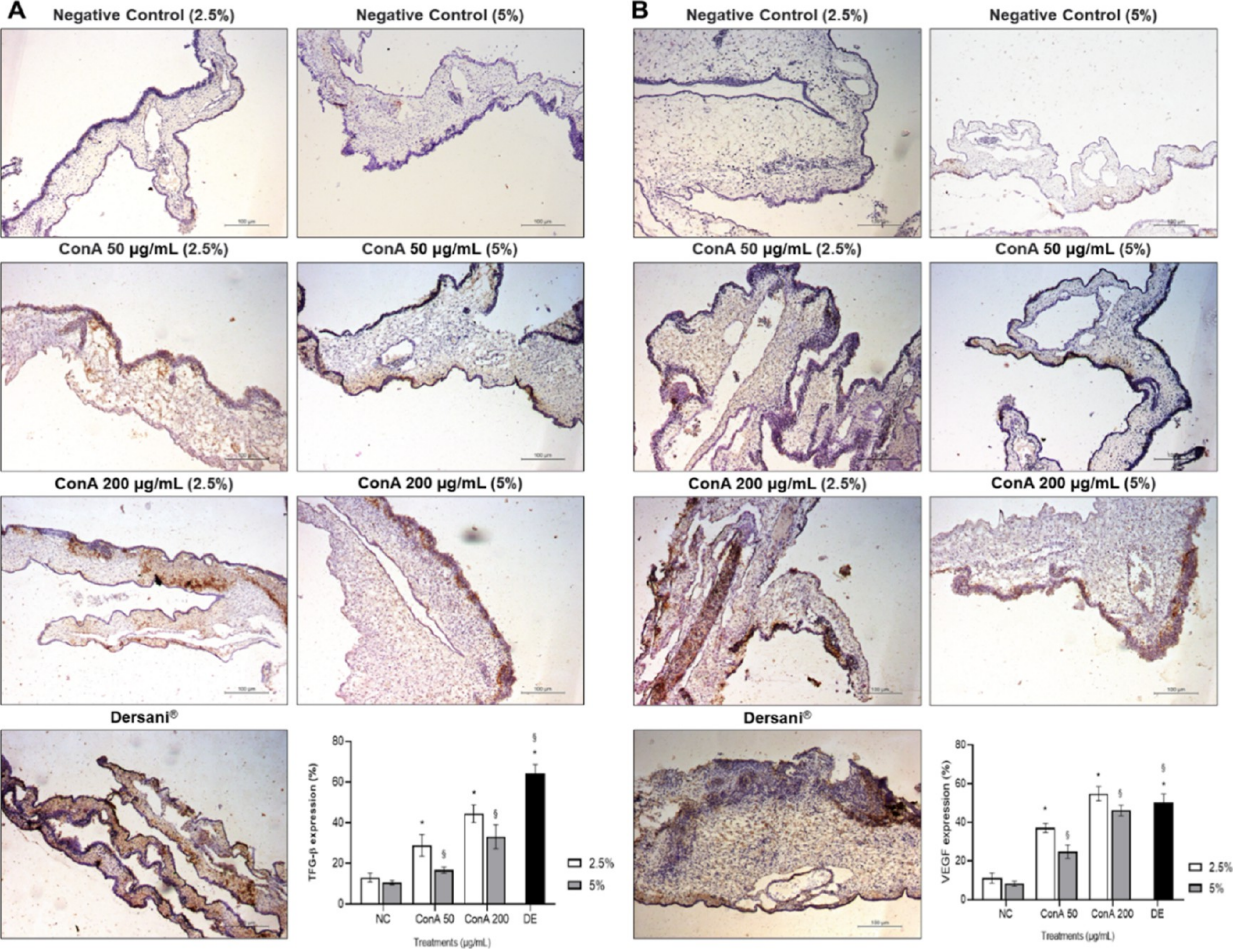
Immunodetection of angiogenic factors in chick embryo chorioallantoic membranes treated with ConA. (A) Immunostaining of vascular endothelial growth factor (VEGF); (B) immunostaining of transforming growth factor-beta (TGF-β). The mean values obtained from each treatment were used to determine the expression (%). * Significant difference compared to the negative control 2.5% (*p* < 0.05); § significant difference compared to the negative control 5% (*p* < 0.05).

We observed, through immunohistochemistry, that films conjugated with ConA present an increase in the expression of the angiogenic factors TGF-β and VEGF. TGF-β plays a crucial role in recruiting inflammatory cells, activating angiogenesis, stimulating extracellular matrix (ECM) deposition, and removing the ECM once the wound has been properly closed. In turn, VEGF is essential for the formation of new blood vessels.^20^

## 4 Conclusion

In this study, alginate/CMC films incorporated with ConA through calcium-mediated cross-links promoted angiogenesis and revascularization of the chorioallantoic membrane, likely due to increased expression of angiogenic factors (TGF-β and VEGF). These films are promising for therapeutic applications that require induction of vascularization, such as wound healing and treatment of ischemic diseases, and may be crucial in the future treatment of diabetic wounds.

## Supplementary Material



## Notes

Figure S1. SDS-PAGE analysis of ConA. Table S1. Assignment of functional groups of alginate and CMC (ConA) film obtained with cross-linking concentrations of 2.5% and 5% (PDF)

## References

[1] Stryker Z. I., Rajabi M., Davis P. J., Mousa S. A. (2019). Evaluation of Angiogenesis
Assays. Biomedicines.

[2] Rajabi M., Mousa S. A. (2017). The Role of Angiogenesis
in Cancer Treatment. Biomedicines.

[3] Ylä-Herttuala S., Bridges C., Katz M. G., Korpisalo P. (2017). Angiogenic
Gene Therapy in Cardiovascular Diseases: Dream or Vision?. Eur. Heart J..

[4] Bisht M., Dhasmana D., Bist S. (2010). Angiogenesis:
Future of Pharmacological
Modulation. Indian J. Pharmacol..

[5] Su W.-H., Cheng M.-H., Lee W.-L., Tsou T.-S., Chang W.-H., Chen C.-S., Wang P.-H. (2010). Nonsteroidal Anti-Inflammatory Drugs
for Wounds: Pain Relief or Excessive Scar Formation?. Mediators Inflammation.

[6] Dodero A., Williams R., Gagliardi S., Vicini S., Alloisio M., Castellano M. (2018). Characterization
of Hyaluronic Acid by Dynamic Light
Scattering and Rheological Techniques. AIP Conf.
Proc..

[7] Kalia, S. ; Avérous, L. Biopolymers; Wiley, 2011.

[8] Morozkina S., Strekalovskaya U., Vanina A., Snetkov P., Krasichkov A., Polyakova V., Uspenskaya M. (2022). The Fabrication of Alginate-Carboxymethyl
Cellulose-Based Composites and Drug Release Profiles. Polymers.

[9] Dodero A., Alloisio M., Vicini S., Castellano M. (2020). Preparation
of Composite Alginate-Based Electrospun Membranes Loaded with ZnO
Nanoparticles. Carbohydr. Polym..

[10] Maity C., Das N. (2022). Alginate-Based Smart Materials and
Their Application: Recent Advances
and Perspectives. Top. Curr. Chem..

[11] Cavada B. S., Osterne V. J. S., Lossio C. F., Pinto-Junior V. R., Oliveira M. V., Silva M. T. L., Leal R. B., Nascimento K. S. (2019). One century
of ConA and 40 years of ConBr research: A structural review. Int. J. Biol. Macromol..

[12] Santos V. F., Araújo A. C. J., Freitas P. R., Silva A. L. P., Santos A. L. E., Matias
da Rocha B. A., Silva R. R. S., Almeida D. V., Garcia W., Coutinho H. D. M., Teixeira C. S. (2021). Enhanced antibacterial
activity of the gentamicin against multidrug-resistant strains when
complexed with *Canavalia ensiformis* lectin. Microb. Pathog..

[13] Roma R. R., Oliveira F. S. A., Fernandes D. G. S., Garcia W., Soares E. N., Costa S. L., Teixeira C. S. (2025). ConA-glutamate
interactions: New
insights into its neuroprotective effect. Int.
J. Biol. Macromol..

[14] Santos A. L. E. D., Souza R. O. S., Barbosa F. E. V., Santos M. H. C. D., Grangeiro Y. A., Martins A. M. C., Santos-Gomes G., Fonseca I. P. D., Silva C. G. L. D., Teixeira C. S. (2024). Concanavalin A,
lectin from *Canavalia ensiformis* seeds has *Leishmania infantum* antipromastigote activity mediated by
carbohydrate recognition domain. Chem. Biol.
Interact..

[15] Fonseca V. J. A., Braga A. L., de Almeida R. S., da Silva T. G., da Silva J. C. P., de Lima L. F., Dos Santos M. H. C., Dos Santos Silva R. R., Teixeira C. S., Coutinho H. D. M., Morais-Braga M. F. B. (2022). Lectins
ConA and ConM extracted from *Canavalia ensiformis* (L.) DC and *Canavalia rosea* (Sw.) DC inhibit planktonic *Candida albicans* and *Candida tropicalis*. Arch. Microbiol..

[16] Vale
de Macedo G. H. R., Chagas V. L., Cruz dos Santos M. H., Costa dos Santos G. D., Bazán J. M. N., Zagmignan A., Carvalho E. M., Mendonça de Miranda R. d. C., Teixeira C. S., Nascimento da Silva L. C. (2022). Development and Characterization
of Alginate-Derived Crosslinked Hydrogel Membranes Incorporated with
ConA and Gentamicin for Wound Dressing Applications. Biochem. Eng. J..

[17] de
Melo Bisneto A. V., de Paiva F. E. A., Fernandes A. S., Roma R. R., Silva L. S., Chiesi G. V., Franchi L. P., Cardoso C. G., Teixeira C. S., Chen-Chen L. (2025). *Dioclea
violacea* lectin exerts pro-angiogenic effects by increasing
VEGF and TNF-α levels via carbohydrate recognition domain. Cytokine.

[18] Véras J. H., Cardoso C. G., Puga S. C., de Melo Bisneto A. V., Roma R. R., Santos Silva R. R., Teixeira C. S., Chen-Chen L. (2022). Lactose-Binding
Lectin from *Vaitarea macrocarpa* Seeds Induces In
Vivo Angiogenesis via VEGF and TNF-α Expression and Modulates
In Vitro Doxorubicin-Induced Genotoxicity. Biochimie.

[19] Li J.-Z., Zhou X.-X., Wu W.-Y., Qiang H.-F., Xiao G.-S., Wang Y., Li G. (2022). Concanavalin
A Promotes Angiogenesis
and Proliferation in Endothelial Cells through the Akt/ERK/Cyclin
D1 Axis. Pharm. Biol..

[20] Ahmad A., Nawaz M. I. (2022). Molecular Mechanism of VEGF and Its
Role in Pathological
Angiogenesis. J. Cell. Biochem..

[21] Laemmli U. K. (1970). Cleavage
of Structural Proteins during the Assembly of the Head of Bacteriophage
T4. Nature.

[22] Auerbach R., Kubai L., Knighton D., Folkman J. A. (1974). Simple Procedure
for the Long-Term Cultivation of Chicken Embryos. Dev. Biol..

[23] Medeiros K. M. d., Araújo E. M., Lira H. d. L., Lima D. d. F., Lima C. A. P. d. (2017). Hybrid Membranes
of Polyamide Applied
in Treatment of Waste Water. Mater. Res..

[24] Bazán J. M. N., Chagas V. L., Silva R. G., Soeiro Silva I. S., Nantes Araujo J. G., Silva L. d. S., Batista K. L. R., Silva R. R. d. S., Correia M. T. d. S., Sousa J. C. d. S., Monteiro C. d. A., Tofanello A., Garcia W. (2023). Development
and Characterization of Alginate-Derived Bioadhesive Films Incorporated
with Anti-Infective Lectins for Application in the Treatment of Oral
Candidiasis. J. Drug Delivery Sci. Technol..

[25] Pavlovčič U., Diaci J., Možina J., Jezeršek M. (2015). Wound Perimeter,
Area, and Volume Measurement Based on Laser 3D and Color Acquisition. Biomed. Eng. Online.

[26] Rouwkema J., Rivron N. C., van Blitterswijk C. A. (2008). Vascularization
in Tissue Engineering. Trends Biotechnol..

[27] Nakipoglu M., Tezcaner A., Contag C. H., Annabi N., Ashammakhi N. (2023). Bioadhesives
with Antimicrobial Properties. Adv. Mater..

[28] Boateng J. S., Matthews K. H., Stevens H. N. E., Eccleston G. M. (2008). Wound Healing
Dressings and Drug Delivery Systems: A Review. J. Pharm. Sci..

[29] Rizky S., Budhijanto, Wintoko J. (2023). Modification
of Bioadhesive Based on Crosslinked Alginate and Gelatin. Mater. Today Proc..

[30] Zhang Z., Li W., Liu Y., Yang Z., Ma L., Zhuang H., Wang E., Wu C., Huan Z., Guo F. (2021). Design of a Biofluid-Absorbing Bioactive Sandwich-Structured
Zn–Si
Bioceramic Composite Wound Dressing for Hair Follicle Regeneration
and Skin Burn Wound Healing. Bioact. Mater..

[31] Rehman S. R., Augustine R., Zahid A. A., Ahmed R., Tariq M., Hasan A. (2019). Reduced Graphene
Oxide Incorporated GelMA Hydrogel Promotes Angiogenesis
For Wound Healing Applications. Int. J. Nanomed..

[32] Nuutila K., Eriksson E. (2021). Moist Wound Healing with Commonly
Available Dressings. Adv. Wound Care.

[33] Senturk
Parreidt T., Müller K., Schmid M. (2018). Alginate-Based Edible
Films and Coatings for Food Packaging Applications. Foods.

[34] Jones V., Grey J. E., Harding K. G. (2006). Wound Dressings. BMJ.

[35] Gontijo, J. F. ; Bierhalz, A. C. K. Membranas De Alginato E Para Liberação De Fármaco: Efeito Da Proporção Polimérica; Blucher: São Paulo, 2018, p 3040.

[36] Reguera J., Urry D. W., Parker T. M., McPherson D. T., Rodríguez-Cabello J. C. (2007). Effect of NaCl on
the Exothermic
and Endothermic Components of the Inverse Temperature Transition of
a Model Elastin-like Polymer. Biomacromolecules.

[37] Medeiros K. M. d., Araújo E. M., Lira H. d. L., Lima D. d. F., Lima C. A. P. d. (2017). Hybrid Membranes of Polyamide Applied
in Treatment of Waste Water. Mater. Res..

[38] de
Melo Bisneto A. V., Fernandes A. S., Velozo Sá V. d. S., Véras J. H., Soares E. T. S., da
Silva Santos A. F., Cardoso C. G., Silveira-Lacerda E. d. P., Carneiro C. C., Chen-Chen L. (2021). Anti-Angiogenic Activity of Azathioprine. Microvasc. Res..

[39] Salama H. E., Abdel Aziz M. S., Alsehli M. (2019). Carboxymethyl Cellulose/Sodium Alginate/Chitosan
Biguanidine Hydrochloride Ternary System for Edible Coatings. Int. J. Biol. Macromol..

[40] Mahheidari N., Kamalabadi-Farahani M., Nourani M. R., Atashi A., Alizadeh M., Aldaghi N., Salehi M. (2024). Biological Study of Skin Wound Treated
with Alginate/Carboxymethyl Cellulose/Chorion Membrane, Diopside Nanoparticles,
and Botox A. NPJ Regen. Med..

[41] Fajardo A. R., Silva M. B., Lopes L. C., Piai J. F., Rubira A. F., Muniz E. C. (2012). Hydrogel Based on
an Alginate–Ca2+/Chondroitin
Sulfate Matrix as a Potential Colon-Specific Drug Delivery System. RSC Adv..

[42] da
Silva Fernandes R., de Moura M. R., Glenn G. M., Aouada F. A. (2018). Thermal,
Microstructural, and Spectroscopic Analysis of Ca2+ Alginate/Clay
Nanocomposite Hydrogel Beads. J. Mol. Liq..

[43] Todica M., Stefan R., Pop C. V., Olar L. (2015). IR and Raman Investigation
of Some Poly­(Acrylic) Acid Gels in Aqueous and Neutralized State. Acta Phys. Pol., A.

[44] Luo S., Lu T., Peng L., Shao J., Zeng Q., Gu J.-D. (2014). Synthesis
of Nanoscale Zero-Valent Iron Immobilized in Alginate Microcapsules
for Removal of Pb­(ii) from Aqueous Solution. J. Mater. Chem. A.

[45] Novosel E. C., Kleinhans C., Kluger P. J. (2011). Vascularization Is the Key Challenge
in Tissue Engineering. Adv. Drug Delivery Rev..

[46] Zilla P., Bezuidenhout D., Human P. (2007). Prosthetic Vascular Grafts: Wrong
Models, Wrong Questions and No Healing. Biomaterials.

[47] Kamoun E. A., Kenawy E.-R. S., Tamer T. M., El-Meligy M. A., Mohy Eldin M. S. (2015). Poly (Vinyl Alcohol)-Alginate Physically
Crosslinked
Hydrogel Membranes for Wound Dressing Applications: Characterization
and Bio-Evaluation. Arab. J. Chem..

[48] Recum A. F. V., Shannon C. E., Cannon C. E., Long K. J., Kooten T. G. V., Meyle J. (1996). Surface Roughness, Porosity, and
Texture as Modifiers
of Cellular Adhesion. Tissue Eng..

[49] Milleret V., Hefti T., Hall H., Vogel V., Eberli D. (2012). Influence
of the Fiber Diameter and Surface Roughness of Electrospun Vascular
Grafts on Blood Activation. Acta Biomater..

[50] Boehringer, J. R. ; Karpowicz, J. ; Mitra, A. ; Radl, C. L. Growth stimulating wound dressing with improved contact surfaces. U.S. Patent 7,951,124 B2, 2011.

[51] Azarpira N., Kaviani M., Sarvestani F. S. (2021). Incorporation of VEGF-and BFGF-Loaded
Alginate Oxide Particles in Acellular Collagen-Alginate Composite
Hydrogel to Promote Angiogenesis. Tissue Cell.

[52] Shen Y., Wang X., Wang Y., Guo X., Yu K., Dong K., Guo Y., Cai C., Li B. (2022). Bilayer Silk
Fibroin/Sodium Alginate Scaffold Promotes Vascularization and Advances
Inflammation Stage in Full-Thickness Wound. Biofabrication.

