# Supplementary material for: Development of Alginate/Carboxymethylcellulose Films Incorporated with Canavalia ensiformis Lectin (ConA) with Angiogenic Properties
Source: ACS Omega. 2025 Nov 3;10(45):54009–21. doi: 10.1021/acsomega.5c05146 (PMC12631320; doi:10.1021/acsomega.5c05146)
Supplement: Supplementary file 1 [file ao5c05146_si_001.pdf]

SUPPLEMENTARY MATERIAL

Development of Alginate/carboxymethylcellulose films incorporated with *Canavalia ensiformis* lectin (ConA) with angiogenic properties

Maria Helena C. Santos [a], Ana Lúcia E. Santos [b], Israel J.M. Santos [c], Renato R. Roma [a], Abel V.M. Bisneto [d], Clever G. Cardoso [d], Bruno A.M. Rocha [a], Lee Chen-Chen [d], Aryane Tofanello [e], Wanius Garcia[e], Luís C.N. Silva [f], Ariane M. S. Santos[g], Edson C. Silva-Filho [g], Claudener S. Teixeira [a,b*].

[a] Department of Biochemistry and Molecular Biology, Federal University of Ceará, Fortaleza, 60451-970, CE, Brazil.
[b] Center for Agricultural Sciences and Biodiversity, Federal University of Cariri, Crato, 63130-025, CE, Brazil.
[c] Department of Biological Chemistry, Regional University of Cariri, Crato, 63105-000, CE, Brazil.
[d] Department of General Biology, Federal University of Goiás, Goiânia, 74001-970, GO, Brazil.
[e] Center for Natural and Human Sciences, Federal University of ABC, 09210-580, SP, Brazil.
[f] Laboratory of Microbial Pathogenesis, CEUMA University, São Luís 65045-380, Brazil.
[g] Interdisciplinary Laboratory of Advanced Materials, Federal University of Piauí, Piauí, 64049-550, Brazil.

*Correspondence to: Claudener Souza Teixeira, Universidade Federal do Cariri - E-mail: claudener.teixeira@ufca.edu.br

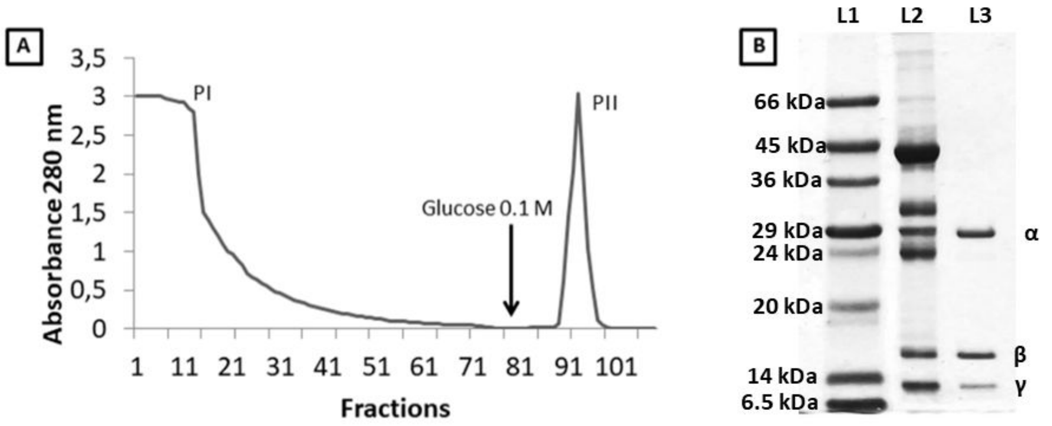

Supplemental Figure 1. ConA purification by affinity chromatography. (A) Chromatogram of *C. ensiformis* crude extract on Sephadex G-50 column. (B) SDS-PAGE of purified ConA. (L1): Molecular weight markers (66–6.5 kDa); (L2): Crude extract of *Canavalia ensiformis*; (L3): Purified ConA.

Table S1. Assignment of functional groups of alginate and CMC (ConA) film obtained with crosslinking concentrations of 2.5% and 5%.

Wavenumber (cm^{-1})	Functional group	Note	Ref.
3220 to 3350	O-H	Hydrogen-bonded O-H stretching vibration of alginate/CMC	19,20
2880 and 2935	C–H	Stretching vibrations of C–H	21
361560 and 1411	COO^- groups	Asymmetric and symmetric stretching vibrations of COO– groups	21
1034 and 924	C–O<br>C–C–H and C–O–H deformation	C–O stretching, incorporating contributions from C–C–H and C–O–H deformation	21
1750 and 1480		Possible interaction between the active groups of ConA and the functional groups of the film, especially the carboxylate groups (COO^-)	22
2880 and 2935	CH_3 group	Rotational vibrations of the CH_3 group are attributed to different skeletal vibrations	36
